# Obesity and Asthma: Physiological Perspective

**DOI:** 10.1155/2013/198068

**Published:** 2013-07-18

**Authors:** Bill Brashier, Sundeep Salvi

**Affiliations:** Chest Research Foundation, Marigold Complex, Kalyani Nagar, Pune, Maharashtra 411014, India

## Abstract

Obesity induces some pertinent physiological changes which are conducive to either development of asthma or cause of poorly controlled asthma state. Obesity related mechanical stress forces induced by abdominal and thoracic fat generate stiffening of the lungs and diaphragmatic movements to result in reduction of resting lung volumes such as functional residual capacity (FRC). Reduced FRC is primarily an outcome of decreased expiratory reserve volume, which pushes the tidal breathing more towards smaller high resistance airways, and consequentially results in expiratory flow limitation during normal breathing in obesity. Reduced FRC also induces plastic alteration in the small collapsible airways, which may generate smooth muscle contraction resulting in increased small airway resistance, which, however, is not picked up by spirometric lung volumes. There is also a possibility that chronically reduced FRC may generate permanent adaptation in the very small airways; therefore, the airway calibres may not change despite weight reduction. Obesity may also induce bronchodilator reversibility and diurnal lung functional variability. Obesity is also associated with airway hyperresponsiveness; however, the mechanism of this is not clear. Thus, obesity has effects on lung function that can generate respiratory distress similar to asthma and may also exaggerate the effects of preexisting asthma.

## 1. Introduction

The obesity prevalence has alarmingly and unprecedentedly increased by more than 3-fold in last three decades, irrespective of socioeconomic status, gender, or age, worldwide [1]. Globally, there are more than 1 billion overweight adults which constitute 300 million clinically obese individuals [1]. 

Obesity is the prime risk factor for various morbidities related to cardiovascular system, metabolic system, and endocrine system. However, in the last 50 years there is a consistent accumulation of evidence to suggest that obesity can significantly impair respiratory well being as well. There is substantial evidence to suggest that obesity enhances the risk of having asthma [2–30] antedate development of asthma [3, 4, 6, 20] and induces asthma worsening amongst preexisting asthmatics [30–33]. The mechanism of this link is however not fully understood and elucidated. 

The current mechanistic understanding of asthma and obesity is a complexity of dietetic factors deficient in antioxidants [34–39], attributes of reduced physical exercise [40, 41], gastroesophageal reflux disease (GERD) [42–44], components of systemic inflammation released from adipose tissue, and mechanical restriction imparted on lung excursions by thoracoabdominal fat [6–8]. Nevertheless, there is a general consensus that obesity increases the metabolic oxygen demand and alongside impairs the lung-diaphragmatic movement. This induces enhanced workload on the lung mechanics to comply with bodily oxygen needs. Hence, obesity is an intriguing clinical state of virtual respiratory distress without respiratory illness. 

In this review we will primarily discuss the physiological changes in the lungs caused by obesity, with its potential role in inducing lung physiology conducive to asthma clinical state.

## 2. Obesity and Asthma Epidemiological Perspective

Obesity has been linked to asthma in various cross-sectional and prospective studies with odds ratio of 1.5–3.5 and relative risk of 1.1–3.5 across adult and paediatric populations [3, 8]. Further, some studies have failed to demonstrate any direct obesity-asthma associations, however, having elucidated positive relation between adipokines (adiposity related inflammatory mediators) and asthma in same populations [45] which indirectly links obesity to asthma. The obesity asthma link is largely attributed to female gender [8, 12–15, 17, 46–48]. However, there is enough evidence to suggest that obesity enhances the risk of asthma in male populations as well [10, 11, 16, 20]. Weight has also demonstrated a dose response association with asthma symptoms [2, 3, 5–8]. In a retrospective study of 143 adults, Akerman et al. [49] demonstrated linear relationship (*r* = 0.40, *P* = 0.0001) between asthma severity and body mass index (BMI). In another study, Varraso et al. [50] had shown that with each 1 kg/m^2^ the asthma severity score changes by 0.183. 

Despite the strong link between asthma and adiposity there are few studies which have failed in elucidating any link between asthma and obesity. This is particularly observed in males and paediatric population [19, 51–56]. Asthma-obesity studies have some pertinent methodological limitations, particularly over reliance on self-reporting of symptoms age and height, and inadequate sample size issues, which could have contributed to the absent obesity-asthma link in some studies. However, it is also important to mention that asthma and BMI demonstrate a U shaped relation [7, 8]. Both extreme high and low BMIs enhance risk of developing asthma [7, 8]. Largely many obesity and asthma studies have compared overweight and obese BMI individuals versus nonobese BMI subjects as control populations. This generates a pertinent risk of also including low BMI high asthma risk population in nonobese BMI comparative groups, which could have negated obesity-asthma associations in some studies.

Additionally, BMI has potential to overdiagnose obesity. BMI defines size of body structure rather than true obesity, which constitutes fatty mass plus body masculinity. The adiposity impacts negatively on the lung functions, while the masculinity impacts positively on the lung functions [57–66]. Physiologically, males have more muscle mass than females. Further, BMI in children may not adequately distinguish increased lean tissue mass from increased fat mass. Therefore, BMI is not always an appropriate marker to study obesity associated diseases in male gender and paediatric populations. This however ensues requirement of actual measure of body adiposity, such as waist/hip ratio (WHR), which reflects central adiposity, subscapular skin-fold thickness, which reflects thoracic adiposity, and skin-fold thickness which reflects general obesity.

Obesity induced lung restriction is one of the prime factors contributing to deranged respiratory mechanics. Therefore, the potential role of body fat distribution in induction of asthma obesity relationship cannot be neglected. It is the abdominal and thoracic fat which interdependently imparts mechanical restriction to the excursions of diaphragm and lungs. The fatty tissue deposits on other bodily sites such as the hips and thighs will not affect the lung movements. In a recent study, Park et al. had shown that overall body fat perse does not contribute to low lung volumes in males, while abdominal obesity significantly reduced FEV1 and FVC irrespective of gender [67]. It is believed that there is more thoracoabdominal fat deposition in females compared to males. This could also be a reason for small airway calibre in females and relatively more obesity predisposition towards asthma in females compared to males. 

Further it has been shown that asthma is more common amongst obese girls who have achieved puberty and menarche earlier. There is also an evidence to suggest that postmenopausal usage of estrogens enhances risk of asthma [25, 50]. Also, progesterone which is known to induce smooth muscle relaxation is decreased in obese states [68, 69]. Such observations cue towards probable role of estrogens-progesterone modulation in obesity in development of asthma in women during puberty. In addition, in a study, Gold et al. followed a group of 9,828 children aged 6–14 years over median period of five years in six US cities to evaluate asthma incidence in the paediatric population [22]. Age of 6–14 years also implies peak period of lung growth. This study showed that during the entry into the study there was an increased risk of a new asthma diagnosis amongst girls with higher BMI, which was not demonstrated in boys. However, there was an increased risk of asthma amongst both boys and girls with largest annual increase in BMI during follow-up period [22]. This study implies that obesity effects during sensitive period of peak lung growth in human life could enhance risk of asthma in future. Also, in women, the age of peak lung growth occurs earlier. Hence, the current presence of obesity may not be enough history to demonstrate associations with asthma. It is important to probe historical trends of obesity, particularly obesity in childhood and adolescence, while probing obesity-asthma associations. This has been largely neglected in most studies. Presence of childhood obesity with current nonobese status could have also contributed to lack of obesity-asthma link.

## 3. Obesity and Physiological Airway Obstruction

High BMI in adults has been associated with reduced FEV1 and FVC [70–72]; however, the FEV1/FVC ratios are minimally reduced due to equivalent decrease in FEV1 and FVC [73, 74]. On the other hand, in children, except for moribund obesity state, increasing BMI has been associated with increasing FEV1 and FVC, however, with reduction in FEV1/FVC ratio [75, 76]. Nevertheless, results of correlation between spirometric lung volumes and body composition have been diverse in each study. These associational variations resulted in a general consensus, that effect of adiposity on lung volume is modest, and both FEV1 and FVC are usually within the normal range in healthy, obese adults and children [77, 78]. Therefore, BMI does not figure in the normal predicted equation of FEV1 and FVC. On the other hand, studies have shown that increasing waist hip ratio, which is a marker of thoracoabdominal obesity, has negative impact on FEV1, FVC, and FEF25-75%, and with every percentage increase in body fat the FEV1 and FVC may decline by 10–15 mL [67, 79]. This suggests that increasing adiposity has potential to induce extra half to three fourth of the normal lung function decline, along with normal lung function decline associated with age and height. This calls for a pertinent consideration of acquisition of body fat mass in development of normal predicted equations of spirometric lung volumes.

Bronchodilator reversibility and diurnal peak flow (PEF) variability are hallmark of asthma diagnosis. There are some studies which show that obesity is associated with bronchodilator reversibility and PEFR variations in populations. In a study by Castro-Rodríguez et al. girls who became obese after age of six had significantly larger height-adjusted postbronchodilator FEV1 response and higher prevalence of peak flow variability than children whose BMI level did not change or decreased after age six [25]. In another study, Hakala et al. showed that with decrease in BMI from 37.2 (3.7) to 32.1 (4.2) kg/m^2^ the diurnal PEF variation declined from 5.5% (2.4) to 4.5% (1.5), and day-to-day variation declined from 5.3% (2.6) to 3.1% (1.3) in adult asthma patients [80]. What is intriguing is that the obesity related asthma lacks eosinophilic inflammation component [81, 82] and is poorly responsive to steroids [83–85] which is unlike allergic asthma. However, there is some evidence to suggest that adipokines can enhance blood IgE levels and airway smooth muscle contraction similar to what has been demonstrated in allergic asthma [86]. 

## 4. Obesity Related Changes in Static Lung Volumes and Its Role in Development of Airway Resistance

**Figure 1:**
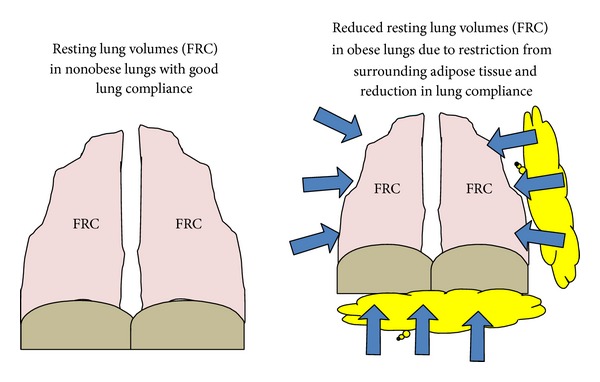
Adiposity resulting reduction in lung compliance and Functional Residual Capacity (FRC).

The abdominal adipose tissue imposes impedance to diaphragmatic contraction and flattening. This generates an inspiratory load and restricting forces on the lungs to optimally inflate and deflate during tidal respiration [87]. The thoracic and subpleural fat may also restrict the ribcage movements imposing additional restrictive forces on the lungs. These alterations in thoracic mechanics cue towards reductions in lung volumes and lung compliance in obese subjects. Adiposity elucidates exponential association with reducing lung compliance [88–90], perhaps due to obesity related increase in pulmonary blood volume, or obesity induced restriction force related closure of collapsible airways and atelectasis, or increased alveolar surface tension [78] (Figure 1). On the other hand, vital capacity (VC) and total lung capacity (TLC) are not significantly altered in obesity. A marginal 20–30% reduction in TLC has been documented with moribund obesity [78, 91–94]. 

**Figure 2:**
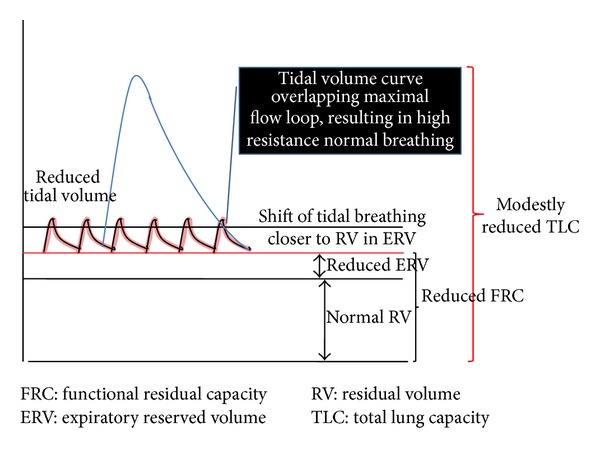
Obese lung tidal breathing volume graph.

Over weight and obesity is known to reduce functional residual capacity (FRC) [91]. Sutherland et al. [95] have shown that with each increase in 1 kg body weight can cause an average reduction of FRC of 28–30 mL, however FRC reduction was primarily associated with thoracic and abdominal adiposity, suggesting a plausible interdependent role of fat distribution in two truncal sites in reducing lung volumes. A reduced FRC in obesity reflects decreased respiratory system compliance (Figure 1). FRC is volume of the air in the lung during end tidal expiration during which the inflationary and deflationary forces in the lungs are at equi-pressure point, and there is no movement of air in the respiratory system. It is a sum of expiratory reserve volume (ERV) and residual volume (RV). RV is well preserved in obesity, or may be slightly elevated [92, 94, 96, 97]. Therefore a reduced FRC in obesity is primarily an attribute of reduced ERV. Studies have shown increasing BMI can generate an exponential reduction in FRC and ERV [88, 91, 92]. Low ERV in obesity suggests occurrence of tidal breathing close to RV in distal high resistance airways, such as noncartilaginous small membranous terminal and respiratory bronchioles and alveolar duct, in obese populations (Figure 2). There is a pertinent linear direct relationship between FRC and airway resistance and inverse linear relation with airway conductance [72, 74, 98, 99]. Studies have shown that high respiratory resistance in obesity normalizes on correction with lung volumes [72, 74, 98, 99]. Ofir et al. [100] showed that obese women had significantly lesser FRC and higher percentage of tidal volume which encroached on maximal flow loops at rest compared to normal weight individuals. However, the same study also showed that exercise induces dynamic increases in FRC which attenuates progressive expiratory flow limitation in obese subject. Impulse oscillometry (IOS) studies in obese subjects elucidate that obesity enhances frequency dependence in the airway resistance and increases lung reactance, suggesting that obesity states primarily affects the small airways and impart elastic load on lung mechanics [101, 102]. Oppenheimer et al. [102] showed that excess body weight resulted in enhanced resistance and reactance in the distal airways which reversed after bariatric surgery. High resistance breathing induced by reduced FRC could increase the asthma symptoms, cause treatment refractoriness, and induce a difficulty to treat asthma state. The noncartilaginous small membranous, terminal and respiratory bronchioles, and alveolar duct collapse at low FRC, suggesting that these airways may close during normal tidal breathing in obesity [90, 103, 104]. Tidal airway closure occurs when the closing volume exceeds the FRC. Therefore, obesity can generate a state of cyclic opening and closing of the airway during normal breathing pattern [105]. This recurrent opening and closing of airways is known to trigger epithelial necrosis and sloughing in the membranous and respiratory bronchioles and rupture of alveolar-bronchiolar attachments and increased leucocytes in the alveolar septa in the animal models [106, 107].

**Figure 3:**
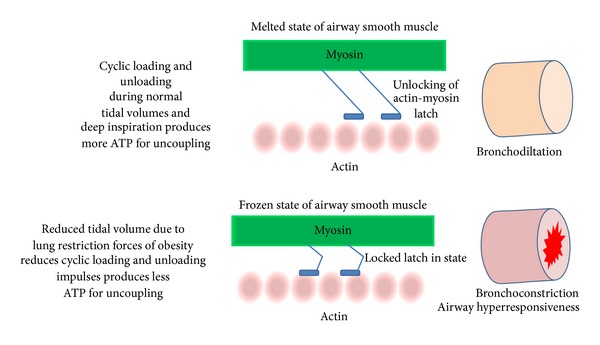
Actin-myosin mechanism of airway narrowing and airway hyperresponsiveness (AHR) in obesity.

A chronic presence of low FRC could also remodel the airways; however, there are no studies which have probed direct changes in the airways of obese humans. There is some evidence to show that despite improvements in lung volumes and reduction in asthma symptoms after weight loss, there is no improvements in volume adjusted airway calibre. This cues towards probable obesity induced remnant of remodelling changes in the airways [78]. However, this can be proved only by carefully designed longitudinal studies. Studies in normal weight subjects have shown that prevention from deep inspiration manoeuvre can increase airway resistance, which does not revert even after reinstating deep inspiration manoeuvre [108–110]. This means that due to chronic restriction in lung movements and reduction in deep inspiration sighs associated with obesity [78], the airway resistance may lose its FRC dependent component over a durational period [97, 111]. The airway smooth muscle has property to adapt to shorter lengths in order to enhance its force generating ability during low lung volume states [112]. This may occur either by plastic adaptation or changes in metabolism of actin and myosin. The smooth muscle contracture apparatus constitutes actin-myosin interaction which promotes a latched-in state [112] (Figure 3). The fluctuation forces of inspiration and expiration imposed by tidal breathing disengages the latched-in state and cause smooth muscle relaxation. Severe obesity is usually associated with reduced tidal volumes with rapid and shallow breathing pattern which further enhances with exercise [78, 91, 94]. Therefore obesity constitutes a state in which the tidal breathing fluctuation forces are inadequate to break the latched-in state of actin and myosin filament. This may result in airway narrowing and increased airway resistance. However, it is also important to mention that in mild-moderate obesity tidal volumes and frequency of deep inspiration remain in normal range [100, 113–115]. Therefore there is a possibility that lung functions may not be altered until late obesity in some cases.

## 5. Obesity and Airway Hyperresponsiveness (AHR)

Airway hyperresponsiveness (AHR) is the cardinal pathophysiology of asthma. Its absence rules out asthma in clinical practise. Unlike obesity and asthma associations, obesity and AHR have not demonstrated any particular epidemiological trends, therefore role of obesity in AHR modulation or vice versa remains largely inconclusive [6, 8]. The effects of weight loss on AHR are also not clear. However there are few large population based cross-sectional and prospective studies which largely indicate a positive link between AHR and obesity [16, 116–118]. There is an evidence to suggest that the incidence and prevalence of AHR increases in obese and underweight population, and its BMI relational graph depicts a U shape association with AHR [16, 117]. Sharma et al. [119] demonstrated that each increase in BMI by 1 kg/m^2^ is associated with a 3.1% increase in AHR risk (95% CI 1.01–1.05). There is also some evidence to suggest that obesity may reduce threshold of AHR to potential asthma triggers. The mouse model studies have shown an exaggerated response to methacholine, which enhances further on ozone exposures, independent of lung size, ventilation rates, and satiety hormones, in genetically obese mouse models (ob/ob) [5, 7]. In the humans Alexeeff et al. [120] have shown that there is an equal decline in FEV1 in obese subjects and subjects with underlying AHR on exposures to ozone. Increased ventilation state triggered by increased oxygen demand associated with obesity could cause inhalation of large doses of air pollution in obese versus nonobese individuals. This then can incur more pollution related lung damage [121]. There is also some indirect evidence to suggest that obesity effects on AHR are more prominent prior to asthma development which diminish postasthma development [6–8]. The possibility of this could be linked to a population based study in 1725 adults by Sood et al. [122] which showed that BMI increases AHR in nonasthmatic subjects and not in asthmatic subjects.

Further, there is no particular gender predilection to the obesity-AHR link [6]. Huang et al. [118] studied AHR in 1459 subjects and showed positive relationship between AHR and BMI only in girls. Chinn et al. [116] evaluated 11,277 European adults and showed that AHR increased with BMI in men but not in women. Jang et al. [123] studied prevalence of methacholine hyperresponsiveness in 677 Korean children and showed an increased AHR in obese boys but not in obese girls. However, data from large population based studies suggest equivalent risk between obesity and AHR amongst both genders.

The mechanism of increased AHR in obesity is not clearly elucidated. There is a possibility of potential involvement of mechanical factors associated with low lung volumes as discussed previously. The animal model studies indicate absence of cellular inflammation in the lungs of unchallenged mice; however, there is increased pulmonary oxidative stress similar to what is seen in asthma [6]. Further, the enhanced AHR in obesity could also be linked to increased production of adipokines such as TNF-*α*, IL6, IL-8, monocyte chemoattractant protein-1, complement proteins, acute phase moieties, leptins and adiponectins from adipocytes and adipose macrophages [6]. It is now increasingly realised that obesity is a state of low grade systemic inflammation which can activate inflammation at sites distant to adipose tissue. Adiponectin is a hormone which is reduced in obesity which has shown to attenuate AHR, eosinophils, and TH2 cells in the lungs of the animal models [6–8]. Another adiposity related hormone, leptin, in concert with other inflammatory agents, has an ability of enhancing AHR. Leptin concentration is 4–6 times higher in severely obese versus nonobese human subjects [124, 125]. However, leptin induces a Th1 type of inflammation rather than Th2 type allergic asthma response [126, 127]. 

There are also various studies suggesting absence of link between obesity and AHR. Here it is important to mention that 8–12% of the general population may have underlying AHR [128]. Also, there are numerous environmental, genetic, and epigenetic influences on AHR which may conceal any additional effect of obesity [6, 129]. This mandates large sample size requirement in order to probe any AHR-determinant associations in epidemiology studies. The overview of obesity AHR studies in humans indicate that most absent-link studies constitute relatively smaller population size [6] compared to the positive link studies [6, 16, 116]. Inadequacy of appropriate sample size could have negated obesity effects on AHR. Further, most negative studies have primarily emerged from Australian and South American regions [52–55]. A study from Chile [55] has even demonstrated an opposite link between obesity and AHR in population (OR 0.93, 95% CI 0.89–0.97). This calls for a possible geographical-regional, environmental, dietetic and genetic heterogeneity factors to this link. Studies in genetically similar animal models have consistently demonstrated positive obesity and AHR associations [5]. Animal model studies have also shown that obesity-AHR has durational component such that mice with longer duration of obesity have higher risk of developing AHR compared to mice with recent onset obesity [29, 129–134].

Further, fall in FEV1 may not be a sensitive measure to study AHR in obesity. Obesity related restrictive forces are more dominant in the smaller airways. Studies have reported small airway closure in the lower (dependent) region in the lungs of obese individuals [78]. This could redistribute the tidal volumes to other airways, which would then receive enhanced tidal volume proportions that could induce dilatation in the airways [135]. FEV1 may not be an appropriate measure to evaluate the complexity of these heterogeneous alterations in patency of bronchopulmonary tree. On the other hand, markers of small airway flow rates (FEF25-75) and small airway resistances could be more relevant in this case. Both adults and children demonstrate obesity related reductions in FEF25-75 compared to lean subjects [5–8]; reduced FEF25-75/FVC has been regarded as marker of increased hyperresponsiveness [136]. Recently, Salome et al. [137] showed that in obese individuals, the only parameter which significantly changed on bronchoprovocation was airway reactance which is a measure of inertive and elastic properties of the lung and has been shown to be the marker of transpulmonary resistance.

## 6. Clinical Implication of Obesity in Asthma

Asthma is primarily managed by inhaled corticosteroids (ICS). However if asthma control is not achieved by ICS, inhaled long-acting b2-agonists (LABA) or other medications may be used as add-on therapy. Intriguingly obesity impinges a relative refractoriness to asthma therapies, and it imposes a state of uncontrolled asthma despite adequate treatments [83, 84]. Boulet et al. [83] had shown that obese asthmatics are less likely than the nonobese asthmatics to achieve asthma control with fluticasone or fluticasone plus salmeterol. However, the cause for steroid unresponsiveness and poor asthma control remains unclear. Further, there is some evidence that treatment unresponsiveness is relatively lesser for leukotriene antagonists in obesity [85]. 

The reason for poor response to asthma treatment can be associated with increased systemic inflammatory state in obesity, but the evidence suggests that the intensity of lung inflammation obese asthmatic is lesser than that of lean asthmatics. Studies have demonstrated an inverse relationship between BMI and pulmonary eosinophils [81, 82] Data from animal models of allergic asthma have shown less eosinophils and BAL lymphocytes in obese mice versus nonobese mice [82].There is also some evidence to suggest reduced mitogen and cytokine T-cell response in obese subjects [138–140]. Therefore it can be assumed that obesity induces a pauci-immune type of asthma. This asthma phenotype demonstrates relative steroid resistance. A recent study has shown that weight reduction with intervention such as bariatric surgery can significantly improve asthma control and treatment responses in asthma, but increase CD4 lymphocytes and their cytokine secreting potential [141]. This suggests that weight reduction can induce an inflammation response in the lungs which may revive steroids responsiveness. However, this needs to be evaluated in proper clinical trials. 

Larger role of mechanical restriction resulting in altered biophysics of lungs to produce a treatment resistant asthma cannot be undermined. The acute and chronic response improvements in asthmatic conditions after weight reduction interventions suggest pertinent role of mechanical restriction forces and altered lung physiology in causing increased treatment refractoriness, poor asthma control, and exacerbation proneness despite adequate treatment in obese populations [142–155]. The treatment modality of obesity related asthma, therefore, should also include weight reduction strategies along with conventional asthma therapy. Treatments which assist in reducing obesity, such as laparoscopic adjustable gastric binding, silastic ring gastric bypass, vertical banded gastroplasty, biliopancreatic diversion with duodenal switch, lap band, weight loss with structured programmes, and low calorie diets have shown to significantly improve quality of life, reduce asthma severity scores, improve asthma symptoms, reduce number of hospitalizations, reduce use of asthma medications, and induce full remissions in obese asthmatic patients [142–155]. However, therapeutic benefits of weight loss have been somewhat equivocal in regards to improvements in AHR in asthma. 

Exercise could be an intriguing intervention in obese asthmatics. Along with potential of reducing weight, exercise may generate deep inspiration and tidal volumes impulses to assist in unlatching actin/myosin latch-in phase and reverse bronchoconstrictions. Reduced physical activity is known to antedate asthma and broncho-hyperresponsiveness [40, 41]. Exercise, on the other hand, can reduce expiratory flow limitation in a dose response manner in obese individuals [100]. Therefore regular exercises along with anti-inflammatory treatments could be an ideal intervention in obese asthma; however, longitudinal studies would be more enlightening.

## 7. Conclusion

Obesity associated restricted lung mechanics induces series of biophysical effects in the lungs which are known to alter lung physiology, such as reduce lung volumes, increased small airway resistance, induce bronchodilator reversibility, induce peak flow variability, and enhance broncho-hyperresponsiveness, which are conducive to development of asthma. The obesity related asthma has poor response to treatment and encompasses gambit of poorly controlled asthma phenotype. However, it is still not known that whether these altered lung physiologies are accompanied by structural pathologies of the lungs particularly airway remodelling. Properly designed studies are mandated to investigate structural changes in the lungs associated with body fat mass and long-term and short-term beneficial effects on the anatomy and physiology of the lungs with reduction in adiposity. 

## References

[1] World Health Organization (WHO) (2013). Obesity and over weight. *Fact Sheet*.

[2] Boulet LP (2013). Asthma and obesity. *Clinical and Experimental Allergy*.

[3] Ford ES (2005). The epidemiology of obesity and asthma. *Journal of Allergy and Clinical Immunology*.

[4] Litonjua AA, Gold DR (2008). Asthma and obesity: common early-life influences in the inception of disease. *Journal of Allergy and Clinical Immunology*.

[5] Shore SA (2007). Obesity and asthma: lessons from animal models. *Journal of Applied Physiology*.

[6] Shore SA (2010). Obesity, airway hyperresponsiveness, and inflammation. *Journal of Applied Physiology*.

[7] Shore SA, Johnston RA (2006). Obesity and asthma. *Pharmacology and Therapeutics*.

[8] Dixon AE, Holguin F, Sood A (2010). An official American Thoracic Society Workshop report: obesity and asthma. *Proceedings of the American Thoracic Society*.

[9] Seidell JC, de Groot LCM, van Sonsbeek JLA (1986). Associations of moderate and severe overweight with self-reported illness and medical care in Dutch adults. *The American Journal of Public Health*.

[10] Arif AA, Delclos GL, Lee ES, Tortolero SR, Whitehead LW (2003). Prevalence and risk factors of asthma and wheezing among US adults: an analysis of the NHANES III data. *European Respiratory Journal*.

[11] Bråbäck L, Hjern A, Rasmussen F (2005). Body mass index, asthma and allergic rhinoconjunctivitis in Swedish conscripts: a national cohort study over three decades. *Respiratory Medicine*.

[12] Nystad W, Meyer HE, Nafstad P, Tverdal A, Engeland A (2004). Body mass index in relation to adult asthma among 135,000 Norwegian men and women. *The American Journal of Epidemiology*.

[13] Chen Y, Dales R, Krewski D, Breithaupt K (1999). Increased effects of smoking and obesity on asthma among female Canadians: the National Population Health Survey, 1994-1995. *The American Journal of Epidemiology*.

[14] Shaheen SO, Sterne JAC, Montgomery SM, Azima H (1999). Birth weight, body mass index and asthma in young adults. *Thorax*.

[15] Mishra V (2004). Effect of obesity on asthma among adult Indian women. *International Journal of Obesity*.

[16] Celedón JC, Palmer LJ, Litonjua AA (2001). Body mass index and asthma in adults in families of subjects with asthma in Anqing, China. *The American Journal of Respiratory and Critical Care Medicine*.

[17] Camargo CA, Weiss ST, Zhang S, Willett WC, Speizer FE (1999). Prospective study of body mass index, weight change, and risk of adult-onset asthma in women. *Archives of Internal Medicine*.

[18] Beckett WS, Jacobs DR, Xinhua YU, Iribarren C, Dale Williams O (2001). Asthma is associated with weight gain in females but not males, independent of physical activity. *The American Journal of Respiratory and Critical Care Medicine*.

[19] Guerra S, Sherrill DL, Bobadilla A, Martinez FD, Barbee RA (2002). The relation of body mass index to asthma, chronic bronchitis, and emphysema. *Chest*.

[20] Beuther DA, Sutherland ER (2007). Overweight, obesity, and incident asthma: a meta-analysis of prospective epidemiologic studies. *The American Journal of Respiratory and Critical Care Medicine*.

[21] Kajbaf TZ, Asar S, Alipoor MR (2011). Relationship between obesity and asthma symptoms among children in Ahvaz, Iran: a cross sectional study. *Italian Journal of Pediatrics*.

[22] Gold DR, Damokosh AI, Dockery DW, Berkey CS (2003). Body-mass index as a predictor of incident asthma in a prospective cohort of children. *Pediatric Pulmonology*.

[23] Gilliland FD, Berhane K, Islam T (2003). Obesity and the risk of newly diagnosed asthma in school-age children. *The American Journal of Epidemiology*.

[24] Mannino DM, Mott J, Ferdinands JM (2006). Boys with high body masses have an increased risk of developing asthma: findings from the National Longitudinal Survey of Youth (NLSY). *International Journal of Obesity*.

[25] Castro-Rodríguez JA, Holberg CJ, Morgan WJ, Wright AL, Martinez FD (2001). Increased incidence of asthmalike symptoms in girls who become overweight or obese during the school years. *The American Journal of Respiratory and Critical Care Medicine*.

[26] He Q-Q, Wong T-W, Du L (2009). Respiratory health in overweight and obese Chinese children. *Pediatric Pulmonology*.

[27] Dong GH, Qian Z, Liu MM (2013). Obesity enhanced respiratory health effects of ambient ai pollution in Chinese children: the Seven North eastern Cities study. *International Journal of Obesity*.

[28] Melanie J, Wijetunga NA, Stepney C (2012). The relationship between asthma and obesity in Urban early adolescents. *Pediatric Allergy Immunology, and Pulmonology*.

[29] Borrell LN, Nguyen EA, Roth LA, Oh SS, Tcheurekdjian H (2013). Childhood obesity and asthma control in the GALA II and SAGE II studies. *The American Journal of Respiratory and Critical Care Medicine*.

[30] Mitchell EA, Beasley R, Björkstén B, Crane J, García-Marcos L, Keil U (2013). The association between BMI, vigorous physical activity and television viewing and the risk of symptoms of asthma, rhinoconjunctivitis and eczema in children and adolescents: ISAAC Phase Three. *Clinical and Experimental Allergy*.

[31] Saint-Pierre P, Bourdin A, Chanez P, Daures J-P, Godard P (2006). Are overweight asthmatics more difficult to control?. *Allergy*.

[32] Peters-Golden M, Swern A, Bird SS, Hustad CM, Grant E, Edelman JM (2006). Influence of body mass index on the response to asthma controller agents. *European Respiratory Journal*.

[33] Mosen DM, Schatz M, Magid DJ, Camargo CA (2008). The relationship between obesity and asthma severity and control in adults. *Journal of Allergy and Clinical Immunology*.

[34] Miedema I, Feskens EJM, Heederik D, Kromhout D (1993). Dietary determinants of long-term incidence of chronic nonspecific lung diseases: the Zutphen study. *The American Journal of Epidemiology*.

[35] Schwartz J, Weiss ST (1990). Dietary factors and their relation to respiratory symptoms. The Second National Health and Nutrition Examination Survey. *The American Journal of Epidemiology*.

[36] Schwartz J, Weiss ST (1994). The relationship of dietary fish intake to level of pulmonary function in the first National Health and Nutrition Survey (NHANES I). *European Respiratory Journal*.

[37] Shahar E, Folsom AR, Melnick SL (1994). Dietary n-3 polyunsaturated fatty acids and smoking-related chronic obstructive pulmonary disease. *The New England Journal of Medicine*.

[38] Peat JK, Salome CM, Woolcock AJ (1992). Factors associated with bronchial hyperresponsiveness in Australian adults and children. *European Respiratory Journal*.

[39] Hodge L, Salome CM, Peat JK, Haby MM, Xuan W, Woolcock AJ (1996). Consumption of oily fish and childhood asthma risk. *Medical Journal of Australia*.

[40] Lucas SR, Platts-Mills TAE (2006). Paediatric asthma and obesity. *Paediatric Respiratory Reviews*.

[41] Shaaban R, Leynaert B, Soussan D (2007). Physical activity and bronchial hyperresponsiveness: european Community Respiratory Health Survey II. *Thorax*.

[42] Harding SM (2001). Gastroesophageal reflux, asthma, and mechanisms of interaction. *The American Journal of Medicine*.

[43] Corley DA, Kubo A (2006). Body mass index and gastroesophageal reflux disease: a systematic review and meta-analysis. *The American Journal of Gastroenterology*.

[44] Jacobson BC, Somers SC, Fuchs CS, Kelly CP, Camargo CA (2006). Body-mass index and symptoms of gastroesophageal reflux in women. *The New England Journal of Medicine*.

[45] Dorevitch S, Conroy L, Karadkhele A, Rosul L, Stacewicz-Sapuntzakis M, Fantuzzi G (2013). Associations between obesity and asthma in a low-income, urban, minority population. *Annals of Allergy, Asthma and Immunology*.

[46] Kronander UN, Falkenberg M, Zetterstrom O (2004). Prevalence and incidence of asthma related to waist circumference and BMI in a Swedish community sample. *Respiratory Medicine*.

[47] Romieu I, Avenel V, Leynaert B, Kauffmann F, Clavel-Chapelon F (2003). Body mass index, change in body silhouette, and risk of asthma in the E3N cohort study. *The American Journal of Epidemiology*.

[48] Huovinen E, Kaprio J, Koskenvuo M (2003). Factors associated to lifestyle and risk of adult onset asthma. *Respiratory Medicine*.

[49] Akerman MJH, Calacanis CM, Madsen MK (2004). Relationship between asthma severity and obesity. *Journal of Asthma*.

[50] Varraso R, Siroux V, Maccario J, Pin I, Kauffmann F (2005). Asthma severity is associated with body mass index and early menarche in women. *The American Journal of Respiratory and Critical Care Medicine*.

[51] Chen Y, Rennie D, Cormier Y, Dosman J (2005). Sex specificity of asthma associated with objectively measured body mass index and waist circumference: the Humboldt study. *Chest*.

[52] Hancox RJ, Milne BJ, Poulton R (2005). Sex differences in the relation between body mass index and asthma and atopy in a birth cohort. *The American Journal of Respiratory and Critical Care Medicine*.

[53] Schachter LM, Peat JK, Salome CM (2003). Asthma and atopy in overweight children. *Thorax*.

[54] Schachter LM, Salome CM, Peat JK, Woolcock AJ (2001). Obesity is a risk for asthma and wheeze but not airway hyperresponsiveness. *Thorax*.

[55] Bustos P, Amigo H, Oyarzún M, Rona RJ (2005). Is there a causal relation between obesity and asthma? Evidence from Chile. *International Journal of Obesity*.

[56] Vázquez-Nava F, Romero JM, Fernández AC (2010). Association between obesity and asthma in preschool Mexican children. *The Scientific World Journal*.

[57] Wannamethee SG, Shaper AG, Whincup PH (2005). Body fat distribution, body composition, and respiratory function in elderly men. *The American Journal of Clinical Nutrition*.

[58] Lazarus R, Gore CJ, Booth M, Owen N (1998). Effects of body composition and fat distribution on ventilatory function in adults. *The American Journal of Clinical Nutrition*.

[59] Santana H, Zoico E, Turcato E (2001). Relation between body composition, fat distribution, and lung function in elderly men. *The American Journal of Clinical Nutrition*.

[60] Cotes JE, Chinn DJ, Reed JW (2001). Body mass, fat percentage, and fat free mass as reference variables for lung function: effects on terms for age and sex. *Thorax*.

[61] Collins LC, Hoberty PD, Walker JF, Fletcher EC, Peiris AN (1995). The effect of body fat distribution on pulmonary function tests. *Chest*.

[62] Lazarus R, Sparrow D, Weiss ST (1997). Effects of obesity and fat distribution on ventilatory function: the normative aging study. *Chest*.

[63] Burchfiel CM, Enright PL, Sharp DS, Chyou P-H, Rodriguez BL, Curb JD (1997). Factors associated with variations in pulmonary function among elderly Japanese-American men. *Chest*.

[64] Chen R, Tunstall-Pedoe H, Bolton-Smith C, Hannah MK, Morrison C (2001). Association of dietary antioxidants and waist circumference with pulmonary function and airway obstruction. *The American Journal of Epidemiology*.

[65] Harik-Khan RI, Wise RA, Fleg JL (2001). The effect of gender on the relationship between body fat distribution and lung function. *Journal of Clinical Epidemiology*.

[66] Canoy D, Luben R, Welch A (2004). Abdominal obesity and respiratory function in men and women in the EPIC-Norfolk study, United Kingdom. *The American Journal of Epidemiology*.

[67] ParK JE, Chung JH, Lee KH, Shin KC (2012). The effect of body composition on pulmonary function. *Tuberculosis and Respiratory Diseases*.

[68] Hernández García IA, Gutiérrez Gutiérrez AM, Gallardo Lozano E (1999). Effect of weight reduction on the clinical and hormonal condition of obese anovulatory women. *Ginecología y obstetricia de México*.

[69] Foster PS, Goldie RG, Paterson JW (1983). Effect of steroids on *β*-adrenoceptor-mediated relaxation of pig bronchus. *British Journal of Pharmacology*.

[70] Schachter LM, Salome CM, Peat JK, Woolcock AJ (2001). Obesity is a risk for asthma and wheeze but not airway hyperresponsiveness. *Thorax*.

[71] Sin DD, Jones RL, Paul Man SF (2002). Obesity is a risk factor for dyspnea but not for airflow obstruction. *Archives of Internal Medicine*.

[72] Zerah F, Harf A, Perlemuter L, Lorino H, Lorino A-M, Atlan G (1993). Effects of obesity on respiratory resistance. *Chest*.

[73] Lazarus R, Sparrow D, Weiss ST (1997). Effects of obesity and fat distribution on ventilatory function: the normative aging study. *Chest*.

[74] Rubinstein I, Zamel N, DuBarry L, Hoffstein V (1990). Airflow limitation in morbidly obese, nonsmoking men. *Annals of Internal Medicine*.

[75] He Q-Q, Wong T-W, Du L (2009). Respiratory health in overweight and obese Chinese children. *Pediatric Pulmonology*.

[76] Tantisira KG, Litonjua AA, Weiss ST, Fuhlbrigge AL (2003). Association of body mass with pulmonary function in the Childhood Asthma Management Program (CAMP). *Thorax*.

[77] Al Ghobain M (2012). The effect of obesity on spirometry tests among healthy non-smoking adults. *BMC Pulmonary Medicine*.

[78] Salome CM, King GG, Berend N (2010). Physiology of obesity and effects on lung function. *Journal of Applied Physiology*.

[79] Chen Y, Rennie D, Cormier YF, Dosman J (2007). Waist circumference is associated with pulmonary function in normal-weight, overweight, and obese subjects. *The American Journal of Clinical Nutrition*.

[80] Hakala K, Stenius-Aarniala B, Sovijarvi A (2000). Effects of weight loss on peak flow variability, airways obstruction, and lung volumes in obese patients with asthma. *Chest*.

[81] Komakula S, Khatri S, Mermis J (2007). Body mass index is associated with reduced exhaled nitric oxide and higher exhaled 8-isoprostanes in asthmatics. *Respiratory Research*.

[82] Johnston RA, Zhu M, Rivera-Sanchez YM (2007). Allergic airway responses in obese mice. *The American Journal of Respiratory and Critical Care Medicine*.

[83] Boulet L-P, Franssen E (2007). Influence of obesity on response to fluticasone with or without salmeterol in moderate asthma. *Respiratory Medicine*.

[84] Anderson WJ, Lipworth BJ (2012). Does body mass index influence responsiveness to inhaled corticosteroids in persistent asthma?. *Annals of Allergy, Asthma and Immunology*.

[85] Peters-Golden M, Swern A, Bird SS, Hustad CM, Grant E, Edelman JM (2006). Influence of body mass index on the response to asthma controller agents. *European Respiratory Journal*.

[86] Shore SA, Schwartzman IN, Mellema MS, Flynt L, Imrich A, Johnston RA (2005). Effect of leptin on allergic airway responses in mice. *Journal of Allergy and Clinical Immunology*.

[87] Naimark A, Cherniack RM (1960). Compliance of the respiratory system and its components in health and obesity. *Journal of Applied Physiology*.

[88] Pelosi P, Croci M, Ravagnan I (1998). The effects of body mass on lung volumes, respiratory mechanics, and gas exchange during general anesthesia. *Anesthesia and Analgesia*.

[89] Pelosi P, Croci M, Ravagnan I, Vicardi P, Gattinoni L (1996). Total respiratory system, lung, and chest wall mechanics in sedated-paralyzed postoperative morbidly obese patients. *Chest*.

[90] Hedenstierna G, Santesson J (1976). Breathing mechanics, dead space and gas exchange in the extremely obese, breathing spontaneously and during anaesthesia with intermittent positive pressure ventilation. *Acta Anaesthesiologica Scandinavica*.

[91] Jones RL, Nzekwu M-MU (2006). The effects of body mass index on lung volumes. *Chest*.

[92] Collins LC, Hoberty PD, Walker JF, Fletcher EC, Peiris AN (1995). The effect of body fat distribution on pulmonary function tests. *Chest*.

[93] Ray CS, Sue DY, Bray G (1983). Effects of obesity on respiratory function. *The American Review of Respiratory Disease*.

[94] Sampson MG, Grassino AE (1983). Load compensation in obese patients during quiet tidal breathing. *Journal of Applied Physiology Respiratory Environmental and Exercise Physiology*.

[95] Sutherland TJT, Goulding A, Grant AM (2008). The effect of adiposity measured by dual-energy X-ray absorptiometry on lung function. *European Respiratory Journal*.

[96] Biring MS, Lewis MI, Liu JT, Mohsenifar Z (1999). Pulmonary physiologic changes of morbid obesity. *The American Journal of the Medical Sciences*.

[97] Watson RA, Pride NB (2005). Postural changes in lung volumes and respiratory resistance in subjects with obesity. *Journal of Applied Physiology*.

[98] Yap JCH, Watson RA, Gilbey S, Pride NB (1995). Effects of posture on respiratory mechanics in obesity. *Journal of Applied Physiology*.

[99] Nicolacakis K, Skowronski ME, Coreno AJ (2008). Observations on the physiological interactions between obesity and asthma. *Journal of Applied Physiology*.

[100] Ofir D, Laveneziana P, Webb KA, O’Donnell DE (2007). Ventilatory and perceptual responses to cycle exercise in obese women. *Journal of Applied Physiology*.

[101] Patel K, Haynes D, Petrini M (2010). Impulse oscillometry (IOS) in obese and lean smokers. *The American Journal of Respiratory and Critical Care Medicine*.

[102] Oppenheimer BW, Macht R, Goldring RM, Stabile A, Berger KI, Parikh M (2012). Distal airway dysfunction in obese subjects corrects after bariatric surgery. *Surgery for Obesity and Related Diseases*.

[103] Hakala K, Mustajoki P, Aittomaki J, Sovijarvi ARA (1995). Effect of weight loss and body position on pulmonary function and gas exchange abnormalities in morbid obesity. *International Journal of Obesity*.

[104] Holley HS, Milic-Emili J, Becklake MR, Bates DV (1967). Regional distribution of pulmonary ventilation and perfusion in obesity. *Journal of Clinical Investigation*.

[105] Milic-Emili J, Torchio R, D’Angelo E (2007). Closing volume: a reappraisal (1967–2007). *European Journal of Applied Physiology*.

[106] D’Angelo E, Pecchiari M, Baraggia P, Saetta M, Balestro E, Milic-Emili J (2002). Low-volume ventilation causes peripheral airway injury and increased airway resistance in normal rabbits. *Journal of Applied Physiology*.

[107] D’Angelo E, Pecchiari M, Saetta M, Balestro E, Milic-Emili J (2004). Dependence of lung injury on inflation rate during low-volume ventilation in normal open-chest rabbits. *Journal of Applied Physiology*.

[108] Skloot G, Permutt S, Togias A (1995). Airway hyperresponsiveness in asthma: a problem of limited smooth muscle relaxation with inspiration. *Journal of Clinical Investigation*.

[109] Moore J, Verburgt LM, King GC, Pare PD (1997). The effect of deep inspiration on methacholine dose-response curves in normal subjects. *The American Journal of Respiratory and Critical Care Medicine*.

[110] King GG, Moore BJ, Seow CY, Paré PD (1999). Time course of increased airway narrowing caused by inhibition of deep inspiration during methacholine challenge. *The American Journal of Respiratory and Critical Care Medicine*.

[111] King GG, Brown NJ, Diba C (2005). The effects of body weight on airway calibre. *European Respiratory Journal*.

[112] Lauzon AM, Bates JHT, Donovan G (2012). A multi scale approach to airway hyperesponsiveness: from molecule to organ. *Frontiers in Physiology*.

[113] Boulet L-P, Turcotte H, Boulet G, Simard B, Robichaud P (2005). Deep inspiration avoidance and airway response to methacholine: influence of body mass index. *Canadian Respiratory Journal*.

[114] Salome CM, Munoz PA, Berend N, Thorpe CW, Schachter LM, King GG (2008). Effect of obesity on breathlessness and airway responsiveness to methacholine in non-asthmatic subjects. *International Journal of Obesity*.

[115] Torchio R, Gobbi A, Gulotta C (2009). Mechanical effects of obesity on airway responsiveness in otherwise healthy humans. *Journal of Applied Physiology*.

[116] Chinn S, Jarvis D, Burney P (2002). Relation of bronchial responsiveness to body mass index in the ECRHS. *Thorax*.

[117] Litonjua AA, Sparrow D, Celedon JC, DeMolles D, Weiss ST (2002). Association of body mass index with the development of methacholine airway hyperresponsiveness in men: the Normative Aging Study. *Thorax*.

[118] Huang S-L, Shiao G-M, Chou P (1999). Association between body mass index and allergy in teenage girls in Taiwan. *Clinical and Experimental Allergy*.

[119] Sharma S, Tailor A, Warrington R, Cheang M (2008). Is obesity associated with an increased risk for airway hyperresponsiveness and development of asthma?. *Allergy, Asthma and Clinical Immunology*.

[120] Alexeeff SE, Litonjua AA, Suh H, Sparrow D, Vokonas PS, Schwartz J (2007). Ozone exposure and lung function: effect modified by obesity and airways hyperresponsiveness in the VA normative aging study. *Chest*.

[121] Bennett WD, Zeman KL (2004). Effect of body size on breathing pattern and fine-particle deposition in children. *Journal of Applied Physiology*.

[122] Sood A, Verhulst SJ, Varma A, Eagleton LE, Henkle JQ, Hopkins-Price P (2006). Association of excess weight and degree of airway responsiveness in asthmatics and non-asthmatics. *Journal of Asthma*.

[123] Jang A-S, Lee JH, Park SW, Shin MY, Kim DJ, Park C-S (2006). Severe airway hyperresponsiveness in school-aged boys with a high body mass index. *Korean Journal of Internal Medicine*.

[124] Maffei M, Halaas J, Ravussin E (1995). Leptin levels in human and rodent: measurement of plasma leptin and ob RNA in obese and weight-reduced subjects. *Nature Medicine*.

[125] Rosenbaum M, Nicolson M, Hirsch J (1996). Effects of gender, body composition, and menopause on plasma concentrations of leptin. *Journal of Clinical Endocrinology and Metabolism*.

[126] Lord GM, Matarese G, Howard JK, Baker RJ, Bloom SR, Lechler RI (1998). Leptin modulates the T-cell immune response and reverses starvation- induced immunosuppression. *Nature*.

[127] Papathanassoglou E, El-Haschimi K, Li XC, Matarese G, Strom T, Mantzoros C (2006). Leptin receptor expression and signaling in lymphocytes: kinetics during lymphocyte activation, role in lymphocyte survival, and response to high fat diet in mice. *Journal of Immunology*.

[128] Kennedy TM, Jones RH, Hungin APS, O’Flanagan H, Kelly P (1998). Irritable bowel syndrome, gastro-oesophageal reflux, and bronchial hyper-responsiveness in the general population. *Gut*.

[129] Johnston RA, Theman TA, Lu FL, Terry RD, Williams ES, Shore SA (2008). Diet-induced obesity causes innate airway hyperresponsiveness to methacholine and enhances ozone-induced pulmonary inflammation. *Journal of Applied Physiology*.

[130] Johnston RA, Theman TA, Shore SA (2006). Augmented responses to ozone in obese carboxypeptidase E-deficient mice. *The American Journal of Physiology*.

[131] Lu FL, Johnston RA, Flynt L (2006). Increased pulmonary responses to acute ozone exposure in obese db/db mice. *The American Journal of Physiology*.

[132] Rivera-Sanchez YM, Johnston RA, Schwartzman IN (2004). Differential effects of ozone on airway and tissue mechanics in obese mice. *Journal of Applied Physiology*.

[133] Shore SA, Rivera-Sanchez YM, Schwartzman IN, Johnston RA (2003). Responses to ozone are increased in obese mice. *Journal of Applied Physiology*.

[134] Shore SA, Williams ES, Zhu M (2008). No effect of metformin on the innate airway hyperresponsiveness and increased responses to ozone observed in obese mice. *Journal of Applied Physiology*.

[135] Winkler T, Venegas JG (2007). Complex airway behavior and paradoxical responses to bronchoprovocation. *Journal of Applied Physiology*.

[136] Litonjua AA, Sparrow D, Weiss ST (1999). The FEF_25-75_/FVC ratio is associated with methacholine airway responsiveness: the normative aging study. *The American Journal of Respiratory and Critical Care Medicine*.

[137] Salome CM, Munoz PA, Berend N, Thorpe CW, Schachter LM, King GG (2008). Effect of obesity on breathlessness and airway responsiveness to methacholine in non-asthmatic subjects. *International Journal of Obesity*.

[138] Tanaka S-I, Isoda F, Ishihara Y, Kimura M, Yamakawa T (2001). T lymphopaenia in relation to body mass index and TNF-*α* in human obesity: adequate weight reduction can be corrective. *Clinical Endocrinology*.

[139] Tanaka S, Inoue S, Isoda F (1993). Impaired immunity in obesity: suppressed but reversible lymphocyte responsiveness. *International Journal of Obesity*.

[140] Sadaf Farooqi I, Matarese G, Lord GM (2002). Beneficial effects of leptin on obesity, T cell hyporesponsiveness, and neuroendocrine/metabolic dysfunction of human congenital leptin deficiency. *Journal of Clinical Investigation*.

[141] Dixon AE, Pratley RE, Forgione PM (2011). Effects of obesity and bariatric surgery on airway hyperresponsiveness, asthma control, and inflammation. *Journal of Allergy and Clinical Immunology*.

[142] Macgregor AMC, Greenberg RA (1993). Effect of surgically induced weight loss on asthma in the morbidly obese. *Obesity Surgery*.

[143] Stenius-Aarniala B, Poussa T, Kvarnström J, Grönlund E-L, Ylikahri M, Mustajoki P (2000). Immediate and long term effects of weight reduction in obese people with asthma: randomised controlled study. *British Medical Journal*.

[144] Johnson JB, Summer W, Cutler RG (2007). Alternate day calorie restriction improves clinical findings and reduces markers of oxidative stress and inflammation in overweight adults with moderate asthma. *Free Radical Biology and Medicine*.

[145] Simard B, Turcotte H, Marceau P (2004). Asthma and sleep apnea in patients with morbid obesity: outcome after bariatric surgery. *Obesity Surgery*.

[146] Sugerman HJ, Sugerman EL, DeMaria EJ (2003). Bariatric surgery for severely obese adolescents. *Journal of Gastrointestinal Surgery*.

[147] Aaron SD, Fergusson D, Dent R, Chen Y, Vandemheen KL, Dales RE (2004). Effect of weight reduction on respiratory function and airway reactivity in obese women. *Chest*.

[148] Ahroni JH, Montgomery KF, Watkins BM (2005). Laparoscopic adjustable gastric banding: weight loss, co-morbidities, medication usage and quality of life at one year. *Obesity Surgery*.

[149] Dixon JB, Chapman L, O’Brien P (1999). Marked improvement in asthma after Lap-Band surgery for morbid obesity. *Obesity Surgery*.

[150] Hall JC, Watts JM, O’Brien PE (1990). Gastric surgery for morbid obesity. The Adelaide study. *Annals of Surgery*.

[151] Murr MM, Siadati MR, Sarr MG (1995). Results of bariatric surgery for morbid obesity in patients older than 50 years. *Obesity Surgery*.

[152] Narbro K, Ågren G, Jonsson E, Näslund I, Sjöström L, Peltonen M (2002). Pharmaceutical costs in obese individuals: comparison with a randomly selected population sample and long-term changes after conventional and surgical treatment: the SOS intervention study. *Archives of Internal Medicine*.

[153] O’Brien PE, Dixon JB, Brown W (2002). The laparoscopic adjustable gastric band (Lap-Band): a prospective study of medium-term effects on weight, health and quality of life. *Obesity Surgery*.

[154] Spivak H, Hewitt MF, Onn A, Half EE (2005). Weight loss and improvement of obesity-related illness in 500 U.S. patients following laparoscopic adjustable gastric banding procedure. *The American Journal of Surgery*.

[155] Dhabuwala A, Cannan RJ, Stubbs RS (2000). Improvement in co-morbidities following weight loss from gastric bypass surgery. *Obesity Surgery*.

